# Hemophagocytic Lymphohistiocytosis Masquerading as Autoimmune Hepatitis

**DOI:** 10.7759/cureus.36543

**Published:** 2023-03-22

**Authors:** Sarosh A Khan, Muhammad Amir

**Affiliations:** 1 Department of Medicine, Integris Baptist Medical Center, Oklahoma City, USA; 2 Department of Hepatology, Integris Baptist Medical Center, Oklahoma City, USA

**Keywords:** hemophagocytic lymphohistiocytosis, aih, liver injury, autoimmune hepatitis, hlh

## Abstract

Hemophagocytic lymphohistiocytosis (HLH) is a complex disease disorder that involves dysregulated activation of the immune system resulting in cytokine storm which can lead to widespread tissue injury. HLH is associated with a mortality rate of 41%. The diagnosis of HLH requires a median of 14 days to reach likely due to a varied range of symptoms and signs the disease can present with. Liver disease and HLH can have a significant overlap. Liver injury itself is frequently noticed in patients with HLH, with more than 50% of patients having elevated aspartate transaminase, alanine transaminase, and bilirubin levels. This case report describes a young individual who had developed intermittent fever, vomiting, fatigue, and weight loss with labs remarkable for elevated transaminases and bilirubin. His initial workup revealed an acute Epstein-Barr virus infection. The patient later presented again with similar signs and symptoms. He underwent a liver biopsy with histopathological features initially concerning for autoimmune hepatitis. However, by engaging a multidisciplinary team, a correct diagnosis was achieved. This case report serves to highlight the increased level of suspicion required to correctly diagnose HLH, especially in the presence of clinical features concerning for autoimmune hepatitis.

## Introduction

Hemophagocytic lymphohistiocytosis (HLH) is a clinical syndrome of dysregulated activation of the immune system resulting in cytokine storm which can lead to widespread tissue injury. Primary HLH is brought upon by genetic mutations affecting the cytotoxic function of immune cells, whereas secondary HLH is triggered by various infectious, autoimmune conditions or malignancies [1]. Over-activation of the immune system brings about a pattern that includes cytopenias, splenomegaly, low fibrinogen, elevated inflammatory markers, and liver/brain injury [2]. The diagnosis of HLH can be challenging as the clinical and laboratory findings can be present in other conditions as well. Additionally, HLH can co-exist with other systemic or organ-specific medical conditions confusing the pathogenesis of injury to a specific organ. We present a case in which HLH-led hepatic injury manifested as autoimmune hepatitis (AIH).

## Case presentation

A 19-year-old male with a medical history of allergic rhinitis presented to the primary care physician in October 2020 with two weeks of intermittent fever, vomiting, and fatigue, as well as 15 lb weight loss in three months. His outpatient labs were significant for thrombocytopenia, elevated transaminases, and bilirubin (aspartate transaminase (AST) 670 U/L, alanine transaminase (ALT) 836 U/L, and total bilirubin 2.9 mg/dL), along with positive Epstein-Barr virus (EBV) IgM and IgG. He was sent to the hospital for further evaluation and management. Upon admission, the patient was ill-appearing with dry oral mucosa, jaundice, palatal petechiae, non-tender cervical lymphadenopathy, and hepatosplenomegaly. On admission, lab work obtained was significant for leukocytosis (white blood cell count 19.3 × 10^3^/mm^3^), thrombocytopenia (platelets 96 × 10^3^/mm^3^), abnormal hepatic parameters (AST 278 U/L, ALT 427 U/L, alkaline phosphatase (ALP) 421 U/L, lactate dehydrogenase (LDH) 885 U/L, total bilirubin 4.8 mg/dL, direct bilirubin 3.9 mg/dL, and international normalized ratio 1.1). His hemoglobin was normal (13.1 g/dL). Tests for viral hepatitis A, B, and C were negative. Ferritin was found to be elevated at 2,184 ng/mL, and the fibrinogen level was 169 mg/dL (reference range = 206-464 mg/dL). Right upper quadrant ultrasound with Doppler showed non-specific gallbladder wall thickening and hepatomegaly. Magnetic resonance cholangiopancreatography (MRCP) was negative for choledocholithiasis. Overall, it was felt that his clinical presentation was due to acute EBV infection and the patient was discharged home after five days of conservative management in the hospital.

The patient presented to the hospital four days after his previous discharge with fever, fatigue, epistaxis, and abdominal discomfort. Labs were significant for anemia (hemoglobin 10.7 g/dL), thrombocytopenia (platelets 113 × 10^3^/mm^3^), low fibrinogen (165 mg/dL), hyperferritinemia (ferritin 2,282 ng/mL) and abnormal hepatic indices (total bilirubin 4.0 mg/dL, AST 639 U/L, ALT 679 U/L, ALP 563 U/L, and LDH 844 U/L). Table 1 presents the laboratory values from the patient’s two inpatient stays. Abdomen/pelvis CT was remarkable for hepatosplenomegaly. Chest CT was remarkable for axillary lymphadenopathy. Differential diagnoses on admission included prolonged EBV infection course, EBV-induced HLH, and lymphoma. He underwent a bone marrow biopsy to evaluate for hemophagocytosis and a lymph node biopsy to exclude lymphoma. The soluble interleukin 2 receptor level was elevated. He was started on dexamethasone after meeting five out of the eight criteria published for HLH, including cytopenia, hepatosplenomegaly, hyperferritinemia, persistent fever, and hypertriglyceridemia (likely triggered secondary to EBV infection) [3]. Bone marrow aspirate pathology later showed normocellular marrow with vague hemophagocytosis, increased CD8-positive T cells consistent with a reactive population, and no evidence of leukemia/lymphoma. Flow cytometry was negative. Lymph node biopsy pathology was consistent with a benign lymph node. The patient’s fever resolved after the initiation of steroids, and he was discharged. Further outpatient workup was negative for hereditary HLH.

**Table 1:** Laboratory values. WBC: white blood cell count; Hb: hemoglobin; Plt: platelet; AST: aspartate transaminase; ALT: alanine transaminase; LDH: lactate dehydrogenase; ASM: anti-smooth muscle; ANA: anti-nuclear antibodies; CMV: cytomegalovirus; EBV: Epstein-Barr virus

Laboratory value	Reference range	First admission	Second admission (10 days later)
WBC	4.6–12.4 K/mm^3^	19.3 K/mm^3^	8.6 K/mm^3^
Hb	12.8–17.4 g/dL	13.1 g/dL	10.7 g/dL
Plt	150–440 K/mm^3^	96 K/mm^3^	113 K/mm^3^
AST	8–42 U/L	278 U/L	639 U/L
ALT	7–40 U/L	427 U/L	679 U/L
Total bilirubin	0.1–1.2 mg/dL	4.8 mg/dL	4.0 mg/dL
LDH	117–278 U/L	885 U/L	844 U/L
Ferritin	10–259 ng/mL	2,184 ng/mL	2,282 ng/mL
Fibrinogen	206–464 mg/dL	169 mg/dL	165 mg/dL
Proteinase 3 Ab	<2.0 = negative	<0.7 U/mL	
Myeloperoxidase	<3.5 = negative	<0.3 U/mL	
ASM titer	<1:20	1:40	
ANA		Negative	
F-actin IgG	0–19 U	26 U	17 U
CMV IgM		Positive	
CMV IgG		Positive	
CMV PCR			<400 IU/mL (not detected)
EBV IgG	0–10.9 U/mL	138 U/mL	
EBV PCR			1,640 copy/mL (detected)
IgM	43–271 mg/dL	480 mg/dL	
IgG	701–1,469 mg/dL	1c,701 mg/dL	

ALT trended down to 68 U/L by the end of November 2020. Dexamethasone was weaned off in about three to four weeks. However, he developed fatigue and jaundice again. Lab workup in early December 2020 revealed rising transaminases (ALT 1,068 U/L and AST 141 U/L) and he was started on a tapering course of prednisone. Workup in the gastroenterology clinic revealed anti-smooth muscle antibody (ASMA) titer 1:80 and F-actin IgG 45 U. A liver biopsy was done in late January 2021 after stopping prednisone for three weeks. His liver biopsy showed mild-to-moderate pan-lobular lymphocytic hepatitis with occasional plasma cells, mild interface hepatitis, scattered small ill-defined granulomata and a prominent background of activated macrophages, without unequivocal hemophagocytosis, mild-to-moderate reticulin collapse with near-complete regeneration/healing, and mild portal/periportal fibrosis (Batts/Ludwig stage 1-2/4). The findings were thought to be concerning for AIH, especially in the setting of recurrent liver injury while off steroids and in the presence of suggestive autoantibodies. The patient was then referred to the transplant hepatology service at our tertiary care center in February 2021. As blood work showed improvement in ALT to 62 U/L while off steroids, a decision was made to observe the patient without steroids. Liver biopsy slides were reviewed at our hospital by an expert pathologist, and it was suggested that the pathology findings likely correlate with HLH rather than AIH due to the presence of endothelialitis; a sinusoidal pattern of activated lymphocytes, macrophages, and suspected hemophagocytosis; and relatively mild interface activity and few plasma cells. His ALT and AST completely normalized by March 2021 and stayed normal afterward without any use of steroids. Eighteen months after his initial episode, ASMA titer normalized as well.

## Discussion

Liver injury is frequently noticed in patients with HLH, with >50% of patients having elevated AST, ALT, and bilirubin levels [4]. Liver disease and HLH can have a significant overlap of clinical manifestations and laboratory abnormalities. Hepatomegaly, encephalopathy, coagulopathy, thrombocytopenia, low fibrinogen, and elevated D-dimer can be attributed to both disease processes. However, HLH is a multisystem disease with cytokine profiles not attributable to AIH. Histopathological findings of the liver in patients with HLH show sinusoidal dilatation, hepatocellular necrosis, endothelialitis, steatosis, and periportal lymphocytic infiltration [4,5].

AIH lacks a diagnostic marker. Diagnosis requires a combination of characteristic biochemical (transaminitis, elevated IgG), specific serological markers, and histological findings while excluding diseases with a similar presentation [6,7]. Serological markers including anti-nuclear antibodies (ANA), ASMA, and anti-liver/kidney microsomal-1 antibodies (anti-LKM-1) are generally checked in patients with AIH [6]. It has been reported that an isolated presence of ANA, ASMA, or anti-LKM-1 can be seen in 49% of AIH patients, while 51% of patients have multiple autoantibodies [7]. The International Autoimmune Hepatitis Group (IAIHG) proposed the diagnostic criteria in 1993 that were revised in 1999 [6]. A simplified scoring system was introduced by the IAIHG in 2008 for application in daily clinical practice [8]. However, the use of the revised and simplified diagnostic criteria was limited due to insufficient validation and lack of accuracy in co-existing liver pathologies [7].

The patient in this report had elevated ASMA titers, transaminitis, and the initial liver biopsy report concerning for AIH. However, his liver injury finally resolved without maintenance immunosuppression, and his transaminases normalized arguing against AIH. Retrospectively, HLH masqueraded as AIH in our patient, at least transiently. Casault et al. reported a case of biopsy-proven AIH who presented with fever and tachycardia with lab findings remarkable for transaminitis and hyperbilirubinemia and was diagnosed with HLH [9]. In another case, AIH was thought to have been a trigger for secondary HLH [10]. Given our experience, it is possible that these two cases had an immunopathological entity similar to our case.

The clinical syndrome associated with HLH along with a contribution from genetic defects, predisposing medical comorbidities, and triggers makes it a complicated disease disorder. Mortality associated with HLH is 41% [11]. Early administration of therapy has been shown to improve survival [12]. However, the diagnosis of HLH is often delayed due to its rarity, complexity, and variable and diverse clinical presentation. In a study by Jagtap et al., a median of 14 days were required to reach a final diagnosis of HLH [5].

## Conclusions

This case report serves to raise awareness among providers in the early diagnosis of HLH, especially in the presence of confusion caused by a clinical course similar to AIH. The diagnosis of HLH is challenging due to the multiple organ systems involved in the disease process. While guidelines exist to aid with diagnosis, it is usually delayed as other etiologies are ruled out. As the mortality rate associated with HLH is high, it requires early detection and prompt treatment for favorable patient outcomes.
